# Genetic Testing and Risk Scores: Impact on Familial Hypercholesterolemia

**DOI:** 10.3389/fcvm.2019.00005

**Published:** 2019-01-29

**Authors:** Ashish Sarraju, Joshua W. Knowles

**Affiliations:** ^1^Division of Cardiovascular Medicine and Cardiovascular Institute, Stanford University, Stanford, CA, United States; ^2^The FH Foundation, Pasadena, CA, United States; ^3^Stanford Diabetes Research Center, Stanford University, Stanford, CA, United States

**Keywords:** polygenic risk scores, genetic testing, familial hypercholesterolemia, genome wide association studies, PCKS9, low-density lipoprotein, homozygous, heterozygous

## Abstract

Familial Hypercholesterolemia (FH) is an inherited lipid disorder affecting 1 in 220 individuals resulting in highly elevated low-density lipoprotein levels and risk of premature coronary disease. Pathogenic variants causing FH typically involve the LDL receptor (*LDLR*), apolipoprotein B-100 (*APOB*), and proprotein convertase subtulisin/kexin type 9 genes (*PCSK9*) and if identified convey a risk of early onset coronary artery disease (ASCVD) of 3- to 10-fold vs. the general population depending on the severity of the mutation. Identification of monogenic FH within a family has implications for family-based testing (cascade screening), risk stratification, and potentially management, and it has now been recommended that such testing be offered to all potential FH patients. Recently, robust genome wide association studies (GWAS) have led to the recognition that the accumulation of common, small effect alleles affecting many LDL-c raising genes can result in a clinical phenotype largely indistinguishable from monogenic FH (i.e., a risk of early onset ASCVD of ~3-fold) in those at the extreme tail of the distribution for these alleles (i.e., the top 8% of the population for a polygenic risk score). The incorporation of these genetic risk scores into clinical practice for non-FH patients may improve risk stratification but is not yet widely performed due to a less robust evidence base for utility. Here, we review the current status of FH genetic testing, potential future applications as well as challenges and pitfalls.

## Introduction

Familial Hypercholesterolemia (FH) is an autosomal dominant genetic disorder characterized by lifelong exposure to highly elevated cholesterol levels, with an estimated prevalence as high as 1 in 200 people (1–3). Those with FH carry a significantly higher risk of premature coronary disease compared to the general population; however, timely diagnosis and initiation of therapeutic strategies can normalize life expectancy (4). Diagnosis is generally made with established clinical criteria such as the Dutch Lipid Clinic Network (DLCN) criteria, along with genetic testing. Beginning with Goldstein and Brown's Nobel prize winning work identifying the low-density lipoprotein receptor gene (*LDLR*), thousands of gene mutations have been implicated as causal of the FH phenotype. The most common variants involve mutations of the *LDLR* gene—estimated to account for >80% of FH cases—followed by mutations of the apolipoprotein B-100 (*APOB*) and proprotein convertase subtulisin/kexin type 9 (*PCSK9*) genes. The identification of a causal FH mutation in an individual is more likely the more severe the presentation. Genetic testing of *LDLR, APOB*, and *PCSK9* in those with extremely high LDL-c (e.g., >250 mg/dl) early onset ASCVD, xanthomas and a family history of severe hypercholesterolemia will reveal a causal mutation >80% of the time, while those with less severe presentations (e.g., absence of xanthomas) may only have a positive genetic test ~50% of the time. In those with only an LDL-c >190 mg/dl as evidence of potential FH, genetic testing may be unrevealing >90% of the time.

The identification of a mutation in FH patients has been shown to improve family based “cascade screening” in many countries (4, 5). In addition, the presence of a mutation in an FH gene conveys a worse prognosis and should prompt consideration of aggressive attempts to lower LDL-c and mitigate ASCVD risk. Khera et al. showed that the risk of ASCVD in an individual with an LDL-c >190 mg/dl PLUS a FH mutation is 22-fold higher than those with an LDL-c <130 mg/dl. In contrast those with an LDL-c >190 mg/dl without an identified FH mutation have a risk “only” 6-fold higher than those with an LDL-c >130 mg/dl (6). This enhanced risk is most likely due to differences in exposure to high LDL-c that may begin sooner after birth and be more severe in those with monogenic mutations.

The inability to identify a pathogenic mutation in a large fraction of phenotypically defined FH patients has prompted intense investigation into to the genetic basis of the severe hypercholesterolemia and early onset ASCVD in so called “genotype negative, phenotype positive” patients. Efforts to identify new FH genes that could account for these phenotypes have been performed in FH cohorts (both in white and non-white populations) (7–9) in population-based studies including those enriched for ASCVD or hypercholesterolemia (6, 10). So far, single genes with large effects rivaling *LDLR, APOB, PCSK9* have not been identified, though a few genes such as *APOE* may be responsible in some cases and biallelic mutations in genes such as *LDLRAP1* can lead to a recessive form of FH (11, 12).

These efforts have highlighted that many phenotypically defined FH patients (with negative standard FH genetic testing) have a “polygenic” predispostion to extremely high LDL-c. Such patients are at the extreme of the distribution for carrying common polymorphisms affecting many loci associated with raised LDL cholesterol (LDL-c) levels. Polygenic risk scores have been developed that can predict LDL-c and ASCVD risk in such individuals (13, 14).

In this review, we describe the genetic basis for FH as well as the impact of genetic testing and polygenic risk scores on the management of FH. We will also briefly discuss homozygous FH (HoFH) where the identification of certain genetic mutations will mostly clearly affect therapeutic decisions.

## What is the Genetic Architecture in Phenotypically Defined FH Patients?

### Monogenic FH

**Figure 1:**
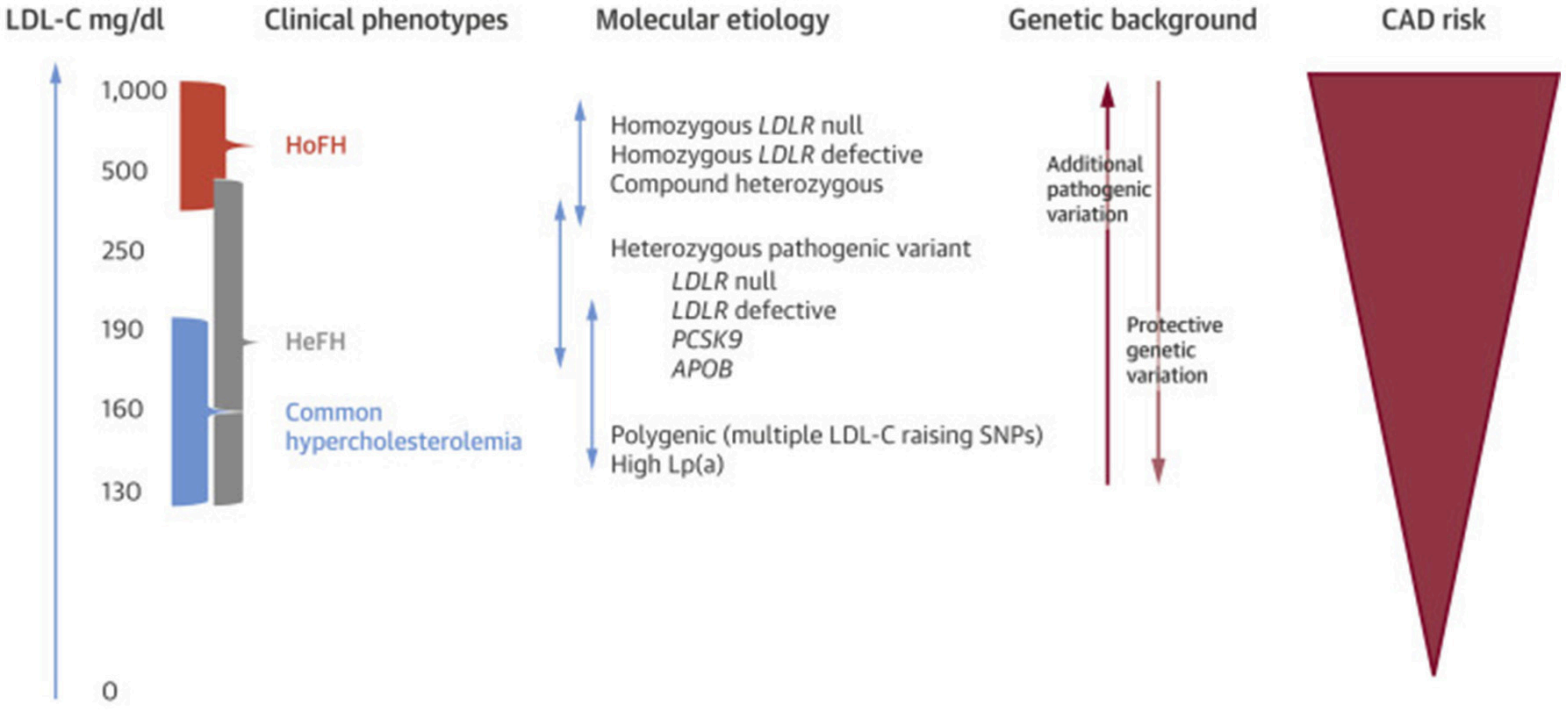
Phenotypic, genotypic, and ASCVD risk spectrum of FH. Lp(a), lipoprotein (a). Other abbreviations as in text. Re-printed with permission from Elsevier (15).

Individuals with FH may carry pathogenic gene variants in one (heterozygous FH or HeFH) or both alleles (homozygous FH or HoFH). Both categories experience lifelong exposure to elevated LDL-c levels and carry an elevated risk of premature coronary disease compared to the general population (4) (Figure 1), though HoFH patients are more severely affected. The most common FH-causing variants are mutations of *LDLR*, followed by mutations of *APOB* and *PCSK9* (Table 1) (4, 16). Other recessive genetic variants that have been associated with comparable hypercholesterolemia syndromes involve*, LDLRAP1, ABCG5*, and *ABCG8* genes (17, 18). There are over 2,000 *LDLR* mutations in the ClinVar database (including those originally deposited in the database from University College London) that result in the FH, the majority of which are single nucleotide substitutions leading to missense mutations (18, 19) though more severe are nonsense mutations that can cause a complete absence of the LDLR. Of note, *LDLR* mutations can affect the protein in a variety of ways but ultimately lead to impaired uptake of circulating LDL-c, and thus severely elevated serum LDL-c levels. Apolipoprotein B-100 is a ligand responsible for LDLR binding during LDL-c uptake, and *APOB* mutations also cause FH through impaired LDL-c uptake (4, 20, 21). In general, *APOB* mutations result in a less severe phenotype compared to *LDLR* mutations (22, 23). Finally, *PCKS9* mutations causative of FH were described in French families with autosomal dominant hypercholesterolemia (24). As PCSK9 is responsible for LDLR degradation in liver cells, FH-causing mutations result in increased PCSK9 activity (gain of function) and increased LDLR degradation (4).

**Table 1:** Overview of common monogenic FH mutations.

**Gene**	**Protein**	**Role of normal protein**	**Type of FH-causative mutation**	**Notes**
*LDLR*	Low-density lipoprotein receptor	Uptake of low-density lipoprotein cholesterol (LDL-c), thus decreasing systemic LDL-c levels	Loss-of-function	60–80% of FH-causative monogenic variants Patients with null *LDLR* mutations may not may not benefit from PCSK9 inhibitors or respond well to statin therapy
*APOB*	Apolipoprotein B-100	Binding of LDL-containing lipoproteins to the LDL receptor	Loss-of-function	Up to 5% of FH-causative monogenic variants (may be higher in some populations)
*PCSK9*	Proprotein convertase subtilisin/kexin 9	Promotes intracellular LDL receptor degradation	Gain-of-function	Up to 3% of FH-causative monogenic variants

*Detailed overview of pathogenic allelic variants for each gene may be found in ClinVar, HGMD (Human Gene Mutation Database), and LOVD (Leiden Open Variation Database) (16)*.

### Polygenic FH

A significant number of patients with clinically diagnosed FH do not have mutations in *LDLR, APOB*, or *PCSK9*. It has been estimated that approximately 40% of patients with “possible FH” (as defined by the DLCN criteria) carry a known pathogenic variant in one of these genes (25). This led to the hypothesis that individuals who carry mutations in multiple common LDL-raising genes may also present with an FH-like phenotype, labeled “polygenic” FH (15). Work identifying polygenic FH initially relied on the power of genome-wide association studies (GWAS). A meta-analysis of GWAS by the Global Lipid Genetic Consortium (GLGC) identified several loci associated with raised LDL-c levels. Talmud et al. demonstrated that individuals who carry multiple LDL-c raising SNPs may also present with significantly elevated LDL-c levels similar to the FH phenotype (26). Subsequently, FH patients without known monogenic mutations were shown to carry an elevated polygenic LDL-c gene score—calculated by incorporating 12 LDL-c raising alleles as identified by the GLGC—compared to healthy controls, suggesting consideration of a polygenic etiology in monogenic mutation-negative FH patients. Khera et al. showed that polygenic risk scores can identify patients in the general population with LDL-c elevations and ASCVD risk similar to that conferred by some monogenic FH variants. They found that approximately 8% of the general population demonstrated at-least 3-fold risk for ASCVD, and 2.3 and 0.5% had 4-fold and 5-fold risk, respectively (14). Natarajan et al. further showed that in individuals with extremely high LDL-c levels, high polygenic risk scores were present in 20% of participants while monogenic mutations were found in 2% (10), supporting the utility of polygenic risk scores in the evaluation of patients with extreme hypercholesterolemia phenotypes.

## How can Genetic Testing and Genetic Risk Scores Inform the Management of ASCVD and FH?

### Genetic Testing in FH

For those with (or potentially with) FH, genetic testing has played a key role in the diagnosis, cascade screening, risk stratification and overall management of FH patients, particularly in many European countries (4, 5, 15). The identification of pathogenic FH mutation in an affected individual allows a definitive diagnosis that can be used for family-based cascade testing. Genetic testing also has implications for risk stratification in FH. Indeed, there is emerging evidence of a relationship between severity of disease presentation and type of *LDLR* mutations. For instance, nonsense *LDLR* mutations may be associated with higher LDL-c levels compared to missense mutations. Additionally, the involved gene may predict disease severity as well; *APOB* and *PCSK9-*related FH phenotypes are generally less severe than *LDLR* phenotypes (22). Monogenic FH has been associated with higher severity of carotid and coronary preclinical atherosclerosis compared to those with a polygenic etiology for hypercholesterolemia (27). Determining the presence of monogenic variants or polygenic FH may also be instructive in formulating cascade screening strategies, as polygenic FH would not be expected to follow the same inheritance pattern as monogenic FH (13).

From a therapeutic standpoint, the most important benefit of genetic testing may be in promoting adherence to lipid lowering therapy. While a pilot study in non-FH patients showed that the use of genetic risk scores did not change lipid levels or adherence to therapy, evidence from several countries suggests that the identification of a pathogenic mutation leads to better adherence to statins and lower LDL-c levels (28–31) in FH patients. Imaging studies such as assessment of coronary artery calcification may hold similar promise for FH-patient motivation; however, younger FH patients may not have significant phenotypic findings (32, 33). Genetic testing results would be available regardless of age, potentially lending genetic testing more uniform clinical applicability for patient motivation. Importantly, the use of traditional ASCVD risk prediction models to make treatment decisions for FH patients is inappropriate, as such models were derived in “average” populations where FH was not adequately represented. Since all FH patients are considered high risk for ASCVD, treatment decisions in FH currently do not depend on risk prediction models. Nevertheless, those FH patients with severe mutations are more likely to suffer earlier onset of ASCVD and may require more aggressive therapy.

An ongoing research question is whether there is a subset of FH patients in the upper echelon of elevated risk who may benefit from early consideration of advanced lipid lowering agents such as PCSK9 inhibitors for primary prevention. The use of polygenic risk scores to assess additive ASCVD risk and potentially identify such extremely high-risk FH patients to prevent morbidity/mortality is an intriguing possibility and warrants further study (14). Talmud et al. have suggested that even in patients with causative mutation-positive FH, polygenic risk scores may help inform additive risk of raised LDL-c levels beyond the FH-causative mutation (13). Ghaleb et al. demonstrated that a high genetic risk score for polygenic hypercholesterolemia may be a marker of phenotype severity in FH patients (34).

ASCVD risk stratification using clinical factors and imaging findings has also been studied. An equation derived from the SAFEHEART registry (Spanish Familial Hypercholesterolemia Cohort Study) using age, sex, history of ASCVD, blood pressure, body-mass index, smoking, LDL-c, and lipoprotein (a) levels demonstrated superior performance for ASCVD prediction in FH patients compared to Framingham and ACC/AHA Omnibus risk prediction tools (35). This equation remains to be validated in other FH populations. A similar idea is captured by the Montreal FH score which, in Canadian FH patients with an *LDLR* mutation, predicted those at high risk for ASCVD events (36). Additionally, subclinical imaging findings such as aortic root or coronary artery calcification have been demonstrated to be independent risk factors for cardiovascular events in patients with FH (32, 33, 37, 38). One limitation of the above studies is that their follow-up periods (median range from 2.7 to 5.5 years) may not have adequately captured the lifetime cumulative ASCVD risk in FH patients. Thus, young FH patients who may not be very high-risk according to the above models (because they have not yet developed clinical/subclinical ASCVD phenotypes) may still be very high-risk over their lifetimes. Additional studies are warranted to examine this question, though this would require large FH cohorts followed for many years. In comparison, genetic variant identification and polygenic risk scores may allow the capture of lifelong risk, and thus, identification of very high-risk FH patients, independent of the presence or severity of clinical/subclinical phenotypes at time of evaluation.

FH genetic testing used to be cost-prohibitive but currently costs are often below $250, which has spurred new lines of enquiry into the cost effectiveness of incorporating genetic testing into FH care in the US. Older data from several European countries and Australia has supported that comprehensive strategies to identify FH patients and perform family-based cascade screening coupled with statin-based therapeutic regimens is highly cost effective (39–43).

There are certain legitimate concerns for the use of FH genetic testing in the US. Many providers are unfamiliar with the tests and educational gaps remain. More importantly, while there is protection against discrimination based on genetic testing for health insurance through the Genetic Information Non-discrimination Act, there is not yet formal protection for life insurance or long-term care insurance, and individuals considering testing need to be made aware of this. All FH genetic testing should be accompanied by comprehensive genetic counseling pre-and post- test. Despite these caveats, there is ample evidence to support that offering genetic testing to potential FH patients should be part of routine clinical care.

### ASCVD Genetic Risk Scores in Non-FH Patients

Traditional risk prediction models for ASCVD are derived from longitudinal study of large populations and framed around clinically identifiable risk factors such as age, sex, diabetes, smoking status, blood pressure, and cholesterol levels. Contemporary prediction tools such as the 2013 ACC/AHA Omnibus calculator are generally employed to make decisions about initiating therapy based on assessment of risk (44). Subsequent efforts to improve risk prediction have also involved incorporation of additional phenotypic information such as coronary artery calcium scores (45). As the role of genetic susceptibility in lifelong risk factor exposure and disease development was made clear, ASCVD risk prediction has begun to focus on genetic risk scores.

The cataloging of several million SNPs in the early 2000s allowed the development of robust GWAS identifying variants associated with clinical outcomes (17, 46). The power and reliability of GWAS have expanded significantly in recent years in part due to large-scale collaboration and data sharing, and there has been increasing focus on studying variants from hundreds of thousands to millions of individuals to develop polygenic risk prediction models. Contemporary studies have drawn from databases with millions of variants, thus facilitating the development of robust genetic risk scores (GRS) which perform favorably compared to traditional risk factors in ASCVD risk prediction, as demonstrated by Khera et al. (46) and Inouye et al. (47). In addition, the establishment of multiple contemporary large-scale biobanks including ethnically diverse patients such as the United Kingdom BioBank and the Million Veterans Program allows the opportunity to derive and validate (or fail to validate) polygenic risk scores with extremely large sample sizes and adequate statistical power (17).

The utility of all risk prediction models must be analyzed in the context of clinical applicability, i.e., what can we do about GRS results for a given patient. In this regard, contemporary evidence suggests that GRS represents actionable information for risk reduction. For instance, Khera et al. demonstrated that lifestyle modification may ameliorate genetic ASCVD risk (48). The efficacy of lifestyle modification in “high-risk” patients as determined by GRS deserves further study. Given the robust performance of genetic scores compared to traditional models of risk prediction, the environment is primed to consider leveraging polygenic risk scores for ASCVD risk prediction in the general population. Studies need to be performed to ascertain whether using GRS (alone or in conjunction with established risk models) results in improved outcomes for patients.

Discussion of the modern role of GRS must involve mention of the rise of direct-to-consumer genetic testing resources through companies such as 23andMe and AncestryDNA. The introduction of consumer-facing genetic testing kits was met with early concern from the medical community. While evidence has accumulated supporting the validity of polygenic scores regarding disease risk, there has also been growing interest from the public in consumer-facing tests. Millions of individuals may have undergone genome-wide genotyping through commercially available tests.

There are certain caveats regarding the use of GRS in clinical settings. We must consider the cost of incorporating genetic risk scores into clinical practice. This includes the potential financial burden to providers and patients to test for and calculate polygenic risk scores at a large scale in any given clinical setting, as well as the time burden of interpreting and communicating these results to patients. Providers may also require additional training and instruction in the analysis of polygenic risk scores. The financial burden of polygenic risk scores may be ameliorated in part by the steady decrease in the cost of genome-wide sequencing—less than $US 100 per person (46). Evidence of outcomes benefit from the use of polygenic risk scores that justifies the potential increase in medical costs is warranted. The role of health insurance with regards to the coverage of genetic testing costs remains unclear, as do the implications of genetic testing results that suggest increased disease risk for future health insurance options. Additionally, the application of polygenic risk scores determined from large scale studies to an individual patient should be performed with some caution. Polygenic risk scores may not accurately estimate effect sizes at the individual level, particularly if risk scores were originally derived from GWAS performed in a different population (49). Examples of populations used to develop polygenic risk scores include the UK Biobank, populations comprised mainly of those with European ancestry, and UK and Belgian patients, which may lead to risk scores with varying degrees of external validity (13, 14, 47). There is concern that the use of GRS which have been mostly derived from and used by white/European populations, can exacerbate health disparities especially until similar GRS can be derived in other race/ethnic groups. It is also possible that individuals possess relevant but unrecognized gene variants, or experience environmental factors that modify their phenotype in a way that is not captured by polygenic risk scores (46). To facilitate evidence-based and informed use of risk scores in clinical settings, prospective studies should validate the use of risk scores in terms of favorable changes in short-term disease management (such as improved medication adherence, improved LDL-c lowering, or improved adherence to lifestyle changes), and long-term clinical outcomes. It is critical that clinicians, patients, government oversight organizations such as the Food and Drug Administration (FDA), and companies collaborate to ensure that these tests are performed and interpreted with the highest quality and in a way that is optimal for patient care.

## Special Considerations for HoFH

Those with homozygous FH present with an accelerated and more severe phenotype compared to those with HeFH, with highly elevated LDL-c levels exceeding 400 mg/dl (16, 50). HoFH patients can present in childhood with dramatic examination findings such as tendon xanthomas and interdigital xanthomas and are at risk for myocardial infarctions or sudden death in the first or second decade of life. Those with HeFH may present with myocardial infarctions as early as the third decade of life. Additionally, HoFH patients are at risk for accelerated valvular disease related to cholesterol deposition. Thus, untreated HoFH may lead to early significant coronary artery disease and valve disease (4). All children of a parent with HoFH will have FH as they will inherit at least one autosomal dominant pathogenic variant (16).

Genetic testing can be a critical aspect of the diagnosis of HoFH, (15). Contemporary FH treatment decisions don't depend on specific mutation status, and are centered on LDL-c lowering–with agents such as statins, bile acid resins, ezetimibe, and PCKS9 inhibitors (4). However, genetic testing may help influence choice of medications and predict response to commonly used medications in FH patients. For instance statins are first-line for both HeFH and HoFH, but they reduce LDL-c levels by only 10–25% in HoFH patients (4). Those HoFH patients with null mutations in *LDLR* may be particularly susceptible to decreased statin efficacy (51). Additionally, HoFH patients who lack LDLR receive no therapeutic benefit from PCSK9 inhibitors (50). Thus, FH patients with *LDLR* null mutations—who generally require aggressive LDL-c reduction early in life—may require prompt consideration of novel therapeutics beyond statins and PCSK9 inhibitors as well as advanced management strategies such as apheresis or liver transplantation. On the other hand, HoFH patients with *LDLR* mutations resulting in defective LDLR activity (not null mutations) may benefit from the initiation of PCSK9 inhibitors (50).

Beyond first-line LDL-c lowering agents, advanced management strategies for HoFH include lipid apheresis, novel therapeutics–lomitapide– and liver transplantation (50). Consideration of these advanced strategies does not depend on results of genetic testing or mutation status beyond the diagnosis of HoFH. Lomitapide is a small molecule inhibitor of microsomal triglyceride transfer protein (MTTP), thus preventing the transfer of lipids between membranes, and is the first-in-class agent to pass phase II clinical trials for HoFH.

## Conclusion

For FH patients, genetic testing for monogenic variants is a key component of diagnosis as well as cascade screening and should be offered as standard-of-care. The rise of polygenic risk scores presents intriguing possibilities for FH management, including facilitating the recognition of polygenic FH in phenotype-positive patients without monogenic mutations, and assessing additive ASCVD risk in monogenic variant-positive patients to identify an “extremely high-risk” population that may benefit from early initiation of advanced FH therapies such as PCKS9 inhibitors for primary prevention. The role of polygenic risk scores on FH management requires further study before implementation in practice, including impact on cost-effectiveness and clinical outcomes. In the future, genetic testing may contribute to the design of individualized FH treatment strategies, particularly in patients with HoFH. In non-FH patients, polygenic risk scores have identified patients in the general population with ASCVD risk comparable to monogenic FH mutation-associated risk. Contemporary GWAS analyzing a broad array of variants from large databases present unique opportunities for future studies to develop and validate polygenic risk scores. The rise of data-sharing initiatives and presence of multiple large biobanks such as the United Kingdom BioBank and the Million Veterans Program will allow the use of very large sample sizes and adequate statistical power for such studies. Moving forward, the clinical role of polygenic risk scores for ASCVD risk estimation and management needs to be further clarified, including effect on clinical outcomes, feasibility for use in routine practice, cost-effectiveness, and effect on insurance coverage options. As genetic testing becomes more affordable and large-scale databases are established, the research environment is primed for the study of polygenic risk scores to expand our understanding of the genetic underpinnings of ASCVD and FH as well as to explore their utility in enhancing risk prediction, diagnosis, and management.

## Author Contributions

AS wrote the first draft of the manuscript along with subsequent revisions. JK conceptualized the article, wrote sections of the manuscript, and contributed meaningfully to revisions.

### Conflict of Interest Statement

The authors declare that the research was conducted in the absence of any commercial or financial relationships that could be construed as a potential conflict of interest.
